# Titanium Dioxide Nanoparticles Increase Superoxide Anion Production by Acting on NADPH Oxidase

**DOI:** 10.1371/journal.pone.0144829

**Published:** 2015-12-29

**Authors:** Rawand Masoud, Tania Bizouarn, Sylvain Trepout, Frank Wien, Laura Baciou, Sergio Marco, Chantal Houée Levin

**Affiliations:** 1 Laboratoire de Chimie Physique, UMR 8000, Université Paris Sud Orsay France; 2 CNRS, UMR 8000, Orsay, France; 3 Institut Curie, Centre de Recherche, Centre Universitaire, Orsay, France; 4 INSERM U1196, Centre Universitaire, Orsay, France; 5 CNRS UMR9187, Centre Universitaire, Orsay, France; 6 Synchrotron SOLEIL, L’Orme des Merisiers, Gif-sur-Yvette, France; Argonne National Laboratory, UNITED STATES

## Abstract

Titanium dioxide (TiO_2_) anatase nanoparticles (NPs) are metal oxide NPs commercialized for several uses of everyday life. However their toxicity has been poorly investigated. Cellular internalization of NPs has been shown to activate macrophages and neutrophils that contribute to superoxide anion production by the NADPH oxidase complex. Transmission electron micrososcopy images showed that the membrane fractions were close to the NPs while fluorescence indicated an interaction between NPs and cytosolic proteins. Using a cell-free system, we have investigated the influence of TiO_2_ NPs on the behavior of the NADPH oxidase. In the absence of the classical activator molecules of the enzyme (arachidonic acid) but in the presence of TiO_2_ NPs, no production of superoxide ions could be detected indicating that TiO_2_ NPs were unable to activate by themselves the complex. However once the NADPH oxidase was activated (i.e., by arachidonic acid), the rate of superoxide anion production went up to 140% of its value without NPs, this effect being dependent on their concentration. In the presence of TiO_2_ nanoparticles, the NADPH oxidase produces more superoxide ions, hence induces higher oxidative stress. This hyper-activation and the subsequent increase in ROS production by TiO_2_ NPs could participate to the oxidative stress development.

## Introduction

Titanium dioxide (TiO_2_) nanoparticles (NPs) are metal oxides NPs manufactured in large quantities and commercialized for several uses because of their high stability, anticorrosive and photocatalytic properties [1]. For example, they are present in household products, plastics industry, electronics, pharmaceutical additives and food colorants [2,3]. In nanomedicine, TiO_2_ NPs are under investigation as useful tools in advanced imaging and nanotherapeutics [4]. TiO_2_ NPs are being explored in cancer diagnosis. They bring many benefits in cancer therapy by absorbing near infrared light [5], and thus being considered as potential photosensitizers for photodynamic therapy [6]. Very promising is the finding that photo-activated nanostructured TiO_2_ exhibited selective cytotoxicity against breast epithelial cancer cells [7]. Furthermore, the physical properties of TiO_2_ NPs make them very interesting products for a use in various skin care and cosmetic products such as sunscreens [8]. TiO_2_ NPs are under investigation as novel treatments for acne vulgaris, atopic dermatitis, hyperpigmented skin lesions, and other non-dermatologic diseases [2,3].

Despite their omnipresence in everyday life, modest research effort has been made in studying their potential adverse effects on living bodies and environment. TiO_2_ NPs can be absorbed into the human body by inhalation, ingestion, and dermal penetration, then they can be distributed to vital organs, including lymph, brain, lung, liver, and kidney [9–11]. TiO_2_ NPs can enter not only in cells, but also mitochondria and nuclei [12]. Most work to date has shown that TiO_2_ NPs toxicity is strongly related to reactive oxygen species (ROS) generation and consequent oxidative stress [12–16].

TiO_2_ NP-mediated ROS responses have been reported to orchestrate a series of pathological events leading to genotoxicity, immunotoxicity, neurotoxicity and carcinogenicity [17,18]. Neutrophils have been shown to be quickly recruited to titanium dioxide areas [19]. Moreover, cellular internalization of TiO_2_ and ZnO NPs has been shown to activate immune cells including macrophages and neutrophils that contribute to ROS production [20–24]. TiO_2_ NPs increased respiratory burst when fish neutrophils were incubated with these NPs [25]. Moreover, they lead to the activation of human ones [23,24]. Recently the same group showed that these nanoparticles enhance the ability of human neutrophils to exert phagocytosis by acting on Syk-dependent signaling pathway [26]. ROS production involves the activation of NADPH oxidase enzymes [22,27], a key player of oxidative stress in immune system cells but also in many other cell types (thyroid, kidney, neurons, and skin) [28–32].

NADPH-oxidase is the only enzyme whose function is to generate superoxide free radicals, which are transformed subsequently into other ROS [33–36]. It is a multi-subunit enzyme complex composed of membrane-bound flavocytochrome *b558* (cyt *b558*), comprising two subunits (Nox2 also known as gp91^phox^, and p22^phox^), present in the membranes phagocytes, and four cytosolic components. Nox2 harbors all the redox carriers (bound FAD, two hemes and the NADPH binding site) that transfer electrons from one side of the membrane cell to the other. The cytosolic components include p47^phox^, p67^phox^, p40^phox^, and a small GTPase Rac1 or Rac2 [37].

In resting phagocytes, the components of the complex exist as separated entities but upon cell activation by pro-inflammatory mediators, the cytosolic subunits undergo posttranslational modifications such as phosphorylation and migrate to the membrane bound cyt *b558* to constitute the activated NADPH-oxidase complex, the only form able to produced superoxide ions [38].

The aim of our study was to investigate the enzymatic behavior of NADPH oxidase in the presence of TiO_2_ NPs and to check if NADPH oxidase could be a pathway involved in ROS generation by TiO_2_ NPs as it has been suggested [27]. We have developed a cell-free system [39–41] that allows controlling the environment, testing and identifying the potential effects of different molecules in various steps of oxidase activation [42,43]. In such cell-free systems, activation is obtained by mixing all proteins with an activator, arachidonic acid (AA). In this study, we have used a construction called trimera, which consisted of the following domains Nter-p47*phox* (amino acids 1-286) linked to the N-ter p67*phox* (amino acids 1-212), and the full length Rac1 Q61L [44]. In a previous paper, we have verified that the rates of production of superoxide anions were similar when the classic cytosolic proteins are replaced by trimera protein to activate the cyt *b558* and also that the dependences of the activity in function of the enzyme activator AA concentration were also found comparable with the cytosolic fractions and the trimera [45]. Thus, the trimera was chosen in order to avoid complications due to some assembly steps and to activate the cyt *b558* in a reproducible manner. We have constantly validated our main conclusions with the separated subunits. We studied not only the effect of TiO_2_ NPs on the function of NADPH oxidase but we also examined their effects on proteins conformations by different methods (fluorescence, synchrotron radiation circular dichroism (SRCD), transmission electron microscope (TEM), dynamic light scattering (DLS)). The use of these combined methods has provided a broad view of how TiO_2_ NPs influence NADPH oxidase functioning and hypotheses about the origin of oxidative stress TiO_2_ NPs dependent.

## Material and Methods

### Materials

Equine heart cytochrome c (cyt *c*), superoxide dismutase from bovine erythrocyte, arachidonic acid (AA), phenylmethanesulfonyl fluoride (PMSF) and Dulbecco phosphate buffer saline (PBS) and standard PBS were from Sigma (Saint Quentin Fallavier, France). Reduced nicotinamide adenine dinucleotide phosphate (NADPH) was from Acros. Ni-sepharose, superdex 75 and Ficoll-Paque Plus were from GE Healthcare, France. Anatase TiO_2_ NPs were a generous gift of Dr Hynd Remita. They were suspended in deionized water (1 mg/mL) and sonicated in an ultra-sound bath for 10 min before use. The experiments were performed in phosphate buffer saline (PBS buffer). It is known that TiO_2_ NPs are affected by the buffer and especially by phosphate [46]. We chose this buffer to be as close as possible to living medium.

### Neutrophil membrane preparation

The neutrophils were prepared from human blood from healthy donors (ESF Paris, France) as described in [47]. Briefly, 500 mL of blood was sedimented in 2% dextran solution for 40 min and centrifuged 400 x g 8 min. Dulbecco PBS was added to the pellets, and then the neutrophils were separated from lymphocytes and the red cells by centrifugation for 30 min at 400 x g on Ficoll solution. The red cells were further eliminated after their lysis by centrifugation for 8 min, 400 g, 4°C. The pellet resuspended in PBS pH 7.4 containing 340 mM sucrose, 7 mM magnesium sulphate, 1 mM PMSF, 0.5 mM leupeptin was sonicated in the 30% pulse mode at power pulses (6) in an ice-cooled beaker 6 times during 10 s with resting time of 1 min between the sonications (sonicator XL, Misonix inc.). Neutrophil membranes and cytosol were separated by centrifugation for 1h30 at 200 000 g at 4°C. The membrane fractions were resuspended, aliquoted and stored at -80°C for further experiments.

### Trimera preparation

The plasmid coding for the trimera was kindly provided by Prof. E. Pick (University of Tel Aviv, Israel). It codes for the Nter-p47*phox* (amino acids 1-286) linked to the N-ter p67*phox* (amino acids 1-212), and the full length Rac1 Q61L [44]. The trimera was expressed and isolated from *E*.*coli BL21-(DE)3-plysS*. Purification of trimera was performed mainly as previously described [45]. Briefly, after a first step through a nickel affinity chromatography, the protein was further purified by size exclusion chromatography; the protein was then dialyzed overnight against a phosphate buffer (100 mM NaCl and 30 mM Na_2_HPO_4_, pH 7.5) and stored at −80°C. The protein concentration was estimated using a NanoDrop2000 spectrophotometer (Thermo scientific, France) and the extinction coefficient of 124,000 mol^-1^ L cm^-1^ at 280 nm (Expasy, Protparam). The purity of all proteins were checked by migration on 10% BisTris-NuPAGE SDS gels (Invitrogen), stained with Coomassie Brilliant Blue and quantified by the ImageJ software.

### Dynamic light scattering measurements

Dynamic light scattering (DLS) experiments were performed to estimate the NPs size. DLS measurements were performed at room temperature on a Malvern NanoZS equipped with a 633 nm laser. Data were collected with a scattering angle of 173°. A range between 2 and 60 μg/mL of TiO_2_ NPs suspensions prepared in PBS or water was tested.

### Transmission electron microscopy measurement

The morphology and size of NPs were also determined by transmission electron microscopy (TEM). The solutions contained 0.5 mg/mL TiO_2_ NPs +/-50 μg/mL trimera and +/-1 mg/mL membrane proteins containing 25μg/mL cyt *b558*. 4 μL of the suspension was deposited onto glow-discharged carbon-coated copper grids and after 1 minute of interaction, the excess of solution was removed with a filter paper (Whatman). As a result, the sample is dried onto the support. Zero-loss (20 eV window) images of TiO_2_ NPs were acquired on field emission gun transmission electron microscope operating at 200 kV (JEOL 2200FS, JEOL LTD^®^).

### Intrinsic fluorescence Assays

Steady-state fluorescence spectra were performed on Fluorolog3- Horiba spectrofluorimeter at 25°C. Various concentrations of TiO_2_ NP suspensions (10- 100 μg/mL) were added as indicated to a final volume of 3 mL of buffer (PBS supplemented with 10 mM MgSO_4_,) containing trimera (5 μg/mL, 60 nM) in a quartz cuvette. The tryptophan fluorescence spectra of trimera were obtained by exciting the samples at 290 nm (2 nm bandwidth) and recorded between 300 to 550 nm (5 nm bandwidth). The excitation wavelength was chosen at 290 nm to optimize the signal to noise ratio and to reduce the contribution of tyrosine residues to the signal [48]. 3 mL of buffer was used as baseline.

### Circular dichroism spectroscopy

Synchrotron radiation circular dichroism (SRCD) spectra were measured on the DISCO beam Line at the synchrotron radiation SOLEIL, Gif/Yvette, France. The calibration was made using a solution of camphorsulphonic acid (CSA). Spectra were measured over the wavelength range from 170 to 260 nm. Three scans were measured and averaged for the samples and the baseline. The averaged baseline was subtracted from the samples and the curves obtained smoothed. SRCD spectra were recorded at 25°C. The solutions contained 1.5 mg/mL (18 μM) trimera +/- 60 μg/mL TiO_2_ NPs, +/- 300 μM AA prepared in 100 mM sodium fluoride; 10 mM sodium phosphate pH 7.0. Spectra are expressed in delta epsilon units, calculated using mean residue weights of 82,681 Da for the trimera. They were fitted using the free software BestSel [49]. Control spectra were recorded with TiO_2_ NPs.

### Measurement of superoxide ion production rates

Superoxide anion production rates were indirectly quantified by the initial rate of cytochrome *c* (cyt *c*) reduction, as previously described [50]. The reaction is the following:

$$Cyt\;c\;{Fe}^{3+}+\;O_{2}^{•-}\;\to \;Cyt\;c\;{Fe}^{2+}+\;O_{2}^{•}$$

Unless indicated, the components of the cell-free system were added as follows: membrane fractions (MF; 2-5 nM cyt *b*_558_), trimera (100-200 nM) and arachidonic acid (40 μM) in 500 μl PBS supplemented with 10 mM MgSO_4_ and incubated for 4 minutes at 25°C in order to allow the NADPH oxidase complex to assemble. The production was initiated by addition of NADPH (250 μM) and the rate of O_2_^-•^ was quantified by the reduction of cyt *c* (50 μM). The rate was measured at 550 nm in a Thermo evolution 500 spectrophotometer, using a molar extinction coefficient (Δε of the reduced *minus* oxidized form of cyt *c*) of 21 mM^-1^ cm^-1^. Control of the production of the O_2_^•-^ species was performed by addition of 50 μg/mL superoxide dismutase (SOD).

## Results

### 3.1 TiO2 NPs size characterization

**Fig 1:**
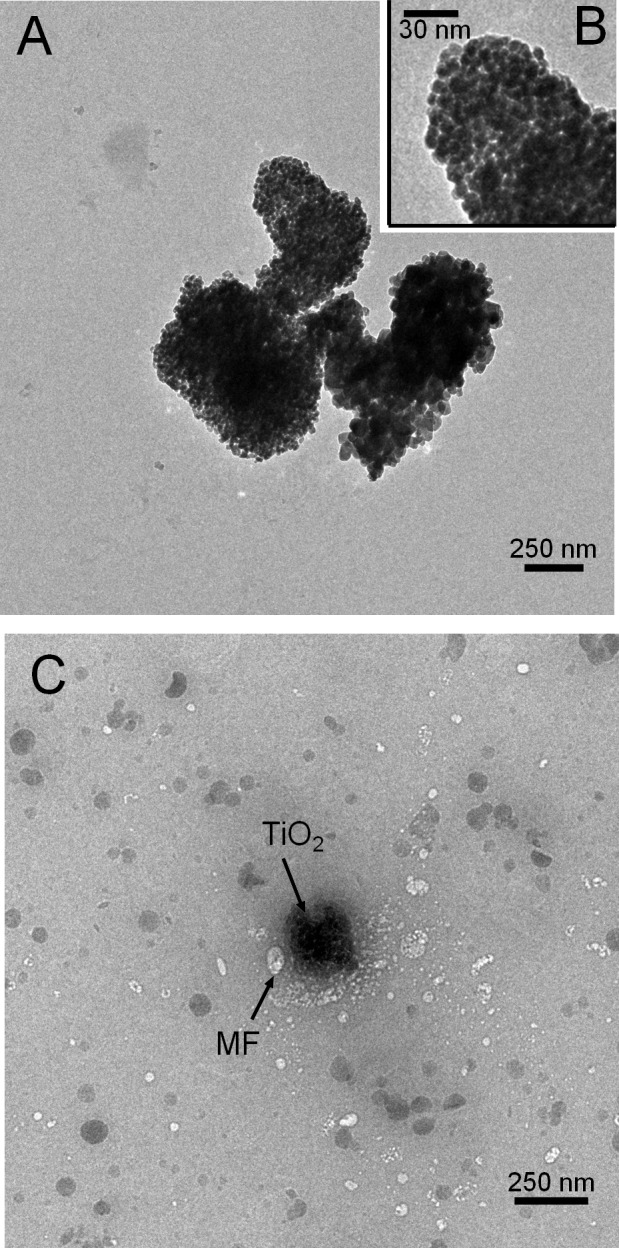
TEM images of (A) 0.5 mg/mL TiO_2_ NPs alone; (B): enlarged view of the cluster; (C) 0.5 mg/mL TiO_2_ NPs with membrane fraction (MF) (25μg/mL cyt *b558*) and 50 μg/mL aggregated trimera;. (The black bar gives the scale: 250 nm for Fig 1A and 1C and 30 nm for Fig 1B). The samples are dried (see materials and methods).

The hydrodynamic size of TiO_2_ NPs in water and PBS was estimated by DLS. The average size of the NPs aggregates in water was about 350 ± 50 nm for the concentration range of 2-80 μg/mL of TiO_2_. This NP aggregate population was predominant (100%) for TiO_2_ NPs concentration lower than 20 μg/mL but another population of larger agglomerates whose sizes were estimated to about ~2000 nm (5%) appeared when TiO_2_ NPs concentration was higher than 20 μg/mL. We noticed also that the size of the NP aggregates in a physiological medium, such as PBS, is similar (460 ± 50 nm) to that in water. By TEM we can also observe particle aggregation (Fig 1A). The aggregates are constituted by particles of about 30± 5 nm (Fig 1B). These results are in accordance with those in the literature where it was shown that TiO_2_ NPs tend to associate to form relatively strongly bonded aggregates or soft agglomerates [17]. TEM images also showed that TiO_2_ NPs are in contact with the membrane fractions (Fig 1C). Moreover, aggregation of TiO_2_ NPs is similar when they were together with the proteins, (Fig 1C). Similarly, DLS measurements showed that the size of TiO_2_ NPs aggregates did not change when MF (0.5μg/mL cyt *b558*) and trimera 18 μg/mL were added to 20 μg/mL TiO_2_ NPs. The concentrations were similar to what we have in the cell free system assays for the measurements of NADPH oxidase activities.

### 3.2 Tryptophan fluorescence of trimera in the presence of TiO2 NPs

**Fig 2:**
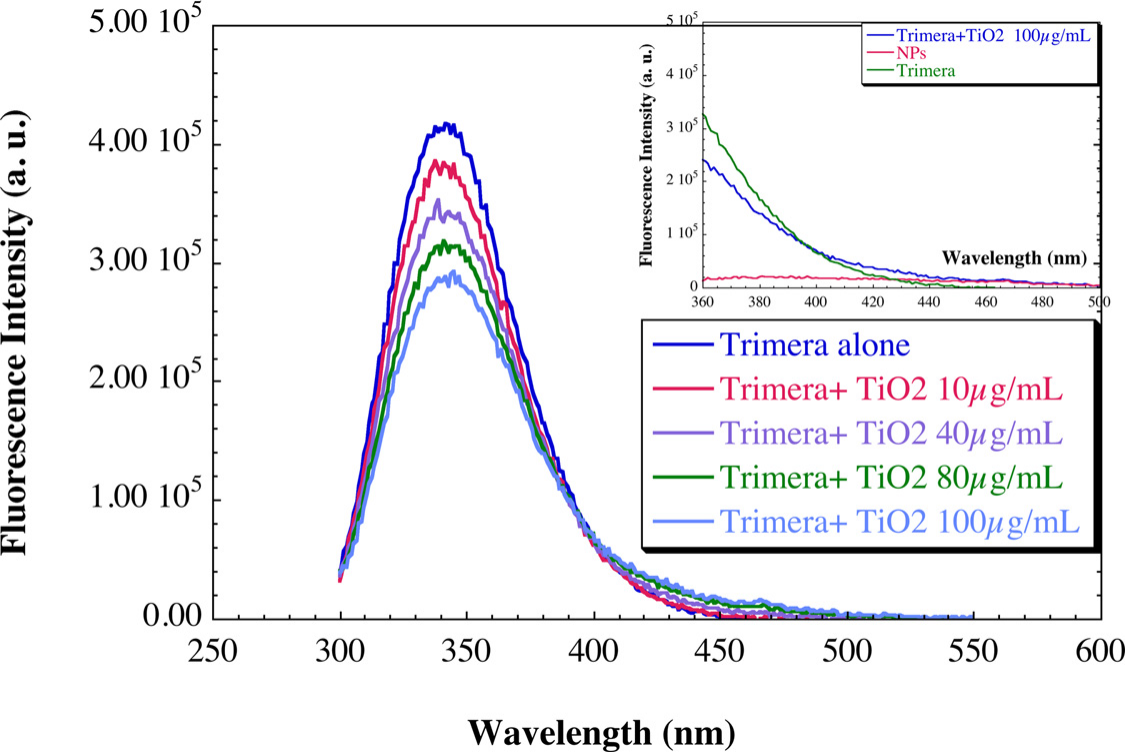
Fluorescence emission spectra of the trimera-TiO_2_ NPs suspensions. The solution contains 5 μg/mL (60 nM) trimera and TiO_2_ NPs at the concentrations of 0, 10, 40, 80 and 100 μg/mL in a final volume of 3 mL of buffer (PBS supplemented with 10 mM MgSO_4_). The emission spectra were measured using an excitation wavelength of 290 nm as described in the Materials and Methods section. Results are representative of at least three independent experiments. In inset: enlargement of the fluorescence spectrum in the region 360-500 nm for three solutions. Fluorescence spectra of 5 μg/mL trimera alone (green), 100 μg/ mL TiO_2_ NPs alone (red) 5 μg/mL trimera in the presence of 100 μg/ mL TiO_2_ NPs (blue).

The conformation changes of the trimera were evaluated by measuring the intrinsic fluorescence spectra of tryptophan residues, before and after addition of TiO_2_ NPs. Trimera contains a total of thirteen tryptophan residues (seven, four and two in the p47*phox*, p67*phox* and Rac portions, respectively). The amplitude of the emission spectrum decreased linearly by the addition of TiO_2_ NPs without any change of the wavelength at the maximum (340 nm) (Fig 2, S1 Fig).The decrease of fluorescence intensity might indicate a quenching due to proximity of TiO_2_ NPs and some tryptophan residues without change in the surrounding of these residues. The intensity of the shoulder at around 440 nm increased concomitantly with the decrease of the intensity of the 340 nm band and is due to emission from TiO_2_ NPs (inset of Fig 2). A similar quenching happens with Trp amino acid in solution with a bathochromic effect on the maximum. This indicates some affinity between Trp and TiO_2_ NPs (S2 Fig)

**Fig 3:**
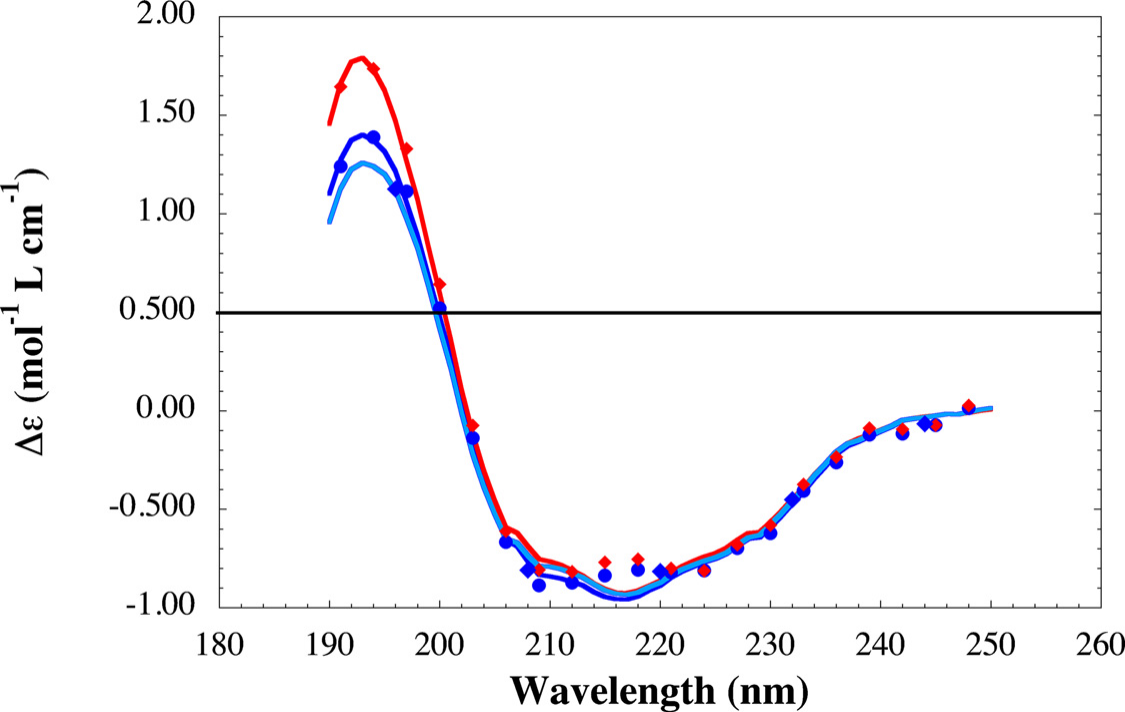
SRCD spectra of trimera alone and in the presence of either TiO2 NPs or AA. SRCD spectra of the trimera (18 μM) alone (Blue) and in the presence of TiO_**2**_ nanoparticles (60 μg/mL) (sky blue) and AA (300μM) (red). The solvent was NaF 100mM /NaPi 10 mM pH 7, 25°C. The points are experimental, the curves are the fits using BeStSel [49].

The eventual changes of the secondary structure due to the NPs were investigated by SRCD spectroscopy. We have recorded the SRCD spectra of 1.5 mg/mL (18μM) trimera in the absence and in the presence of 60 μg/mL TiO_2_ NPs and 300 μM AA (Fig 3). In S1 Table are gathered the percentages of α-helices and β-sheets obtained by fitting the spectra with the Bestsel software [49]. Analysis of the SRCD spectra of the trimera indicates that this chimeric protein is mostly in random coil (ca. 40%) and that the content of helices is very low (3-4%) (S1 Table). Although it was supposed that the addition of an amphiphile like AA would induce larger changes in the structure of the cytosolic proteins, [48,51] we observe only slight modifications of trimera secondary structure upon addition of AA. Similarly, only slight changes in the structure are observed with NPs. There is a loss of α-helixes and an increase of the disorder (S1 Table).

Altogether these results show that the interaction between NPs and trimera, indicated by fluorescence quenching, have no big consequence on the secondary structure of trimera.

### 3.3 Effects on the functionality

#### 3.3.1 Effects of TiO2 NPs on the NADPH oxidase activity

**Fig 4:**
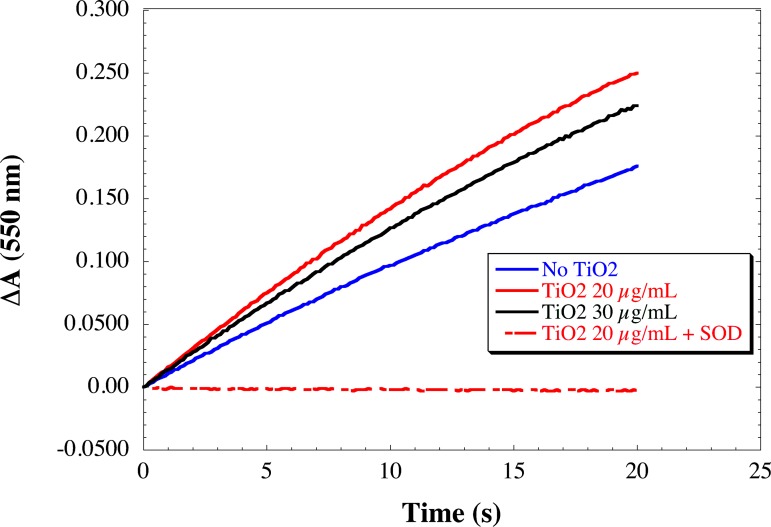
Kinetics of superoxide anion production in presence of TiO_2_ NPs. Neutrophil membrane fractions (5 nM cyt *b558*) and trimera 200 nM were incubated together in the presence of 40 μM AA and (0, 20, 30 μg/mL) TiO_2_ NPs. The production was initiated by addition of NADPH (250 μM) and the rate of O_2_^-•^ was quantified by the reduction of cyt *c* (50 μM). Control was performed by the addition of 50 μg/mL SOD. (on the fig: 20μg/mL TiO_2_ in the presence of SOD). The initial rates of production of superoxide are the following: 92.0±0.3, 134.0±0.5, 119.2±0.4 mol O_2_^•-^/s/Mol Cyt *b*_558_ for TiO2 NPs 0, 20, 30 μg/m respectively.

First, we have checked that TiO_2_ NPs alone did not reduce cyt *c* (data not shown), which means that in these conditions, NPs by themselves do not produce superoxide ions. In order to investigate the effect of NPs on the NADPH oxidase, the rate of superoxide anion production was measured upon addition of TiO_2_ NPs in the cell free assay conditions previously optimized with trimera [45] (Fig 4). The initial slope of the kinetic curve is equal to the rate of superoxide anion formation. This rate was faster in the presence of TiO_2_ NP. The identification of O_2_^-•^ was performed by addition of 50 μg/mL SOD.

**Fig 5:**
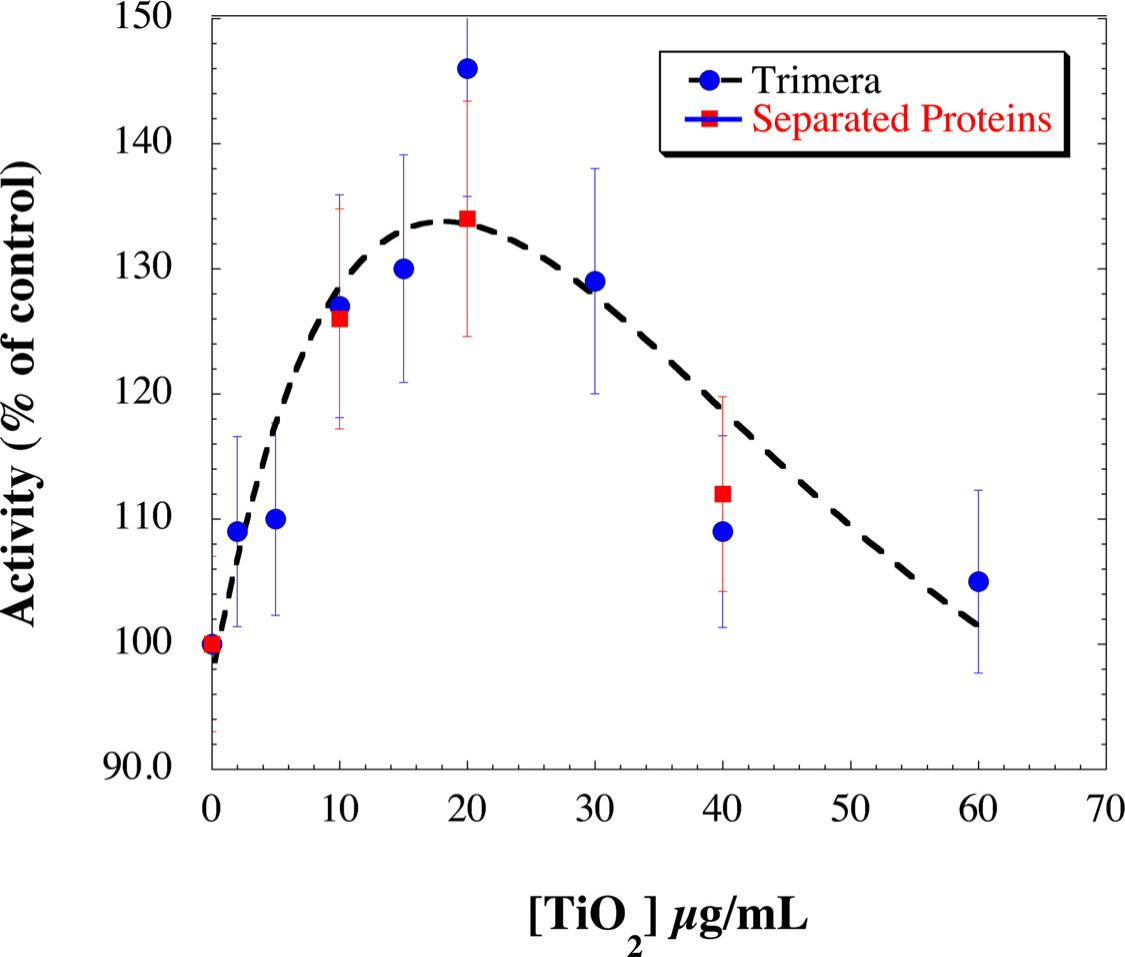
Dependence of NADPH oxidase activity as a function of TiO_2_ NPs concentration. Neutrophil membrane fractions (5 nM cyt *b558*) and trimera 200 nM (blue dots) or the cytosolic subunits (p67^phox^ 200 nM, p47^phox^ 260 nM and Rac 580 nM) (red squares) were incubated together in the presence of 40 μM AA and TiO_2_ NPs. Oxidase activities were expressed as the percent of activity measured in the absence of TiO_2_ NPs (90 mol O_2_^.-^/s/mol cyt *b558*), and determined as 100%. Points are an average of 3 independent measurements. The dotted curve is a visual fit for both systems.

The activity of the complex was investigated in parallel with either the trimera or the mix of cytosolic proteins p47^phox^, p67^phox^ and Rac. All components were incubated together with TiO_2_ NPs (2-60) μg/mL and 40 μM AA. The rate of superoxide anion production in the absence of NPs was considered as 100% of NADPH oxidase activity. No major difference was noticed between the trimera and the cytosolic proteins (Fig 5). In both cases we clearly observed an increase in the NADPH oxidase activity in the presence of NPs. The curves of Fig 5 exhibit a bell shape profile with a maximum (140% of the reference) at around 20 μg/mL of TiO_2_ NPs. For higher concentrations of TiO_2_ NPs (> 20 μg/mL), the rate returned close to the activation level of the control. This result indicates that TiO_2_ NPs potentiate the NADPH oxidase activity. The activity remains constant for concentrations higher than 40 μg/mL probably due to some aggregations of NPs at higher concentrations.

**Fig 6:**
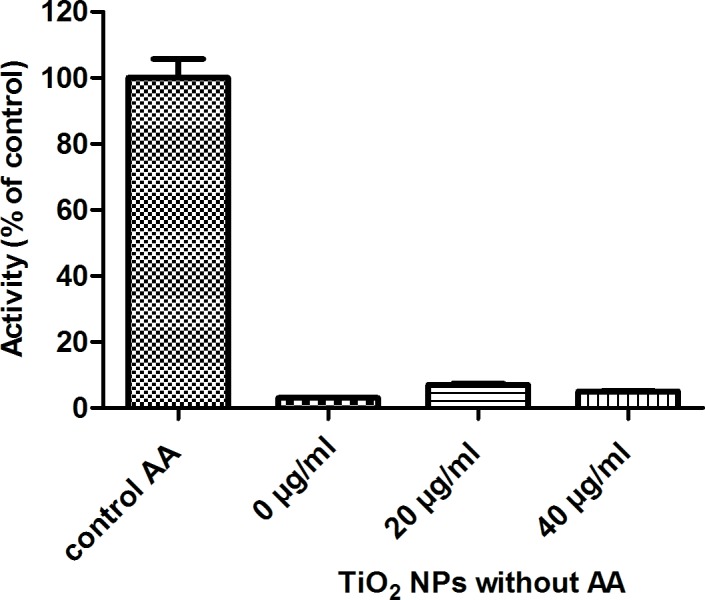
Dependence of NADPH oxidase activity as a function of TiO_2_ NPs concentrations in the absence of arachidonic acid. Membrane fractions (4 nM cyt *b558*) with trimera 200 nM were incubated 4 min in the presence of 0, 20 or 40 μg/mL TiO_2_ NPs. Control experiment representing 100% (83 mol O_2_^•−^/s/ mol cyt *b558*) of the activity was realized in presence of 40 μM AA and in absence of TiO_2_ NPs. The rates of superoxide production were measured as described in Materials and Methods. Data are the average of 3 independent measurements.

Thus, we further questioned whether TiO_2_ NPs alone (20 or 40 μg/mL) could activate the NADPH oxidase complex and thus replace AA as activator (Fig 6). Almost no NADPH oxidase activity (5 ± 2%) was detected with NPs instead of AA (control). Comparable results were obtained using the separated subunits where a maximum activity of 4 ± 2% of AA- dependent activity was reached (data not shown).

**Fig 7:**
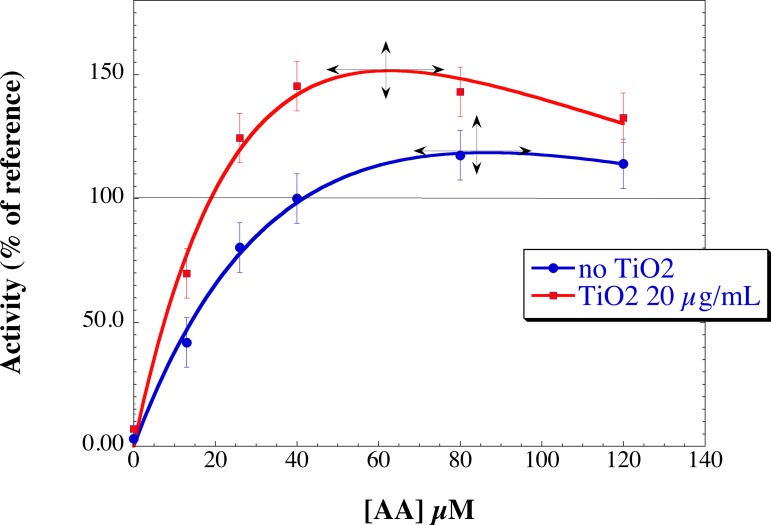
Effect of TiO_2_ NPs on the AA-dependent activation profile. Neutrophil membrane fractions and trimera were incubated together in the presence of different concentration of AA. The TiO_2_ NPs concentration was as follow, blue dots: no TiO_2_ NPs; red squares: 20 μg/mL TiO_2_ NPs. Oxidase activities were expressed as the percent of activity measured in the presence of 40 μM AA (85 mol O_2_^.-^/s/mol cyt *b558*) set as 100%. The curves are visual fits of the experimental points and the maxima have been indicated by crosses. The rate of O_2_^•−^ production was measured as described in Materials and Methods.

Since TiO_2_ NPs cannot be considered as activating molecules, the significant increase in the rate of O_2_^•−^ production with NPs might be due to an indirect effect on the optimized oxidase condition by disturbing the optimal AA concentration. We therefore investigated the effect of TiO_2_ NPs on the AA activation profile. To probe this effect, we performed titrations of the oxidase activity vs. AA concentration in the absence and in the presence of 20 μg/mL TiO_2_ NPs added after arachidonic acid (Fig 7). The rate of production with 40 μM AA alone (the concentration used as reference in this paper), was considered as 100%. In agreement with the above -mentioned results, in the presence of TiO_2_ NPs, the O_2_^•−^ production rate was higher on the full range of AA concentrations. Both curves exhibited bell-shapes as usual but the optimal concentration of AA was lower (ca. 62 μM) in the presence than in the absence (ca. 90 μM) of NPs. Similar results were obtained when NPs were added before AA (data not shown).

#### 3.3.2 Effect of TiO2 NPs addition at different sequences of cell free system assay

**Fig 8:**
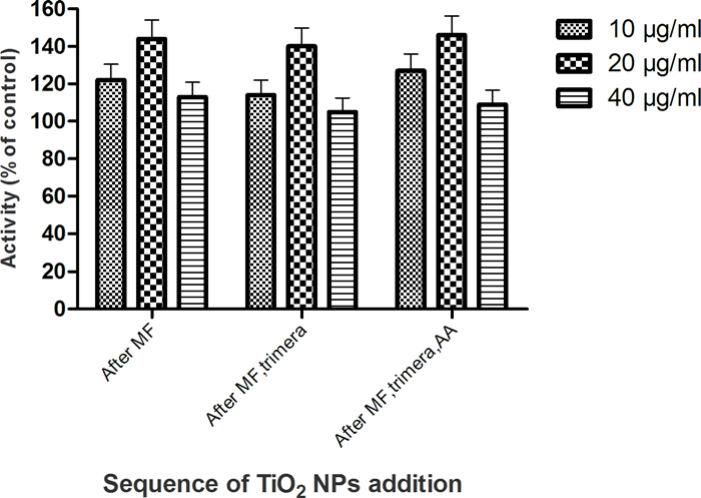
Effect of TiO_2_ NPs as a function of its sequence of addition in the cell free system. Neutrophil membrane fractions (4 nM cyt *b558*) and 200 nM trimera were incubated together in the presence of 40 μM AA and TiO_2_ NPs (10, 20, 40 μg/mL). TiO_2_ NPs was added to the solution either after the membrane fractions or after the membrane fractions and trimera or after the membrane fractions, trimera and AA. Oxidase activity was expressed as the percent of activity measured in the absence of TiO_2_ NPs (84 mol O_2_^.-^/s/mol cyt *b558*) set as 100%. Results are presented as the mean±SD of 3 independent experiments.

**T**o examine whether TiO_2_ NPs have effects on specific steps of the assembly, several concentration of TiO_2_ NPs (10, 20, 40 μg/mL) were added at different times: (i) to the membrane fractions alone before mixing to the cytosolic subunits; (ii) to mixed membrane fractions and trimera; (iii) to the membrane fractions plus trimera plus AA (Fig 8). Regardless the stages at which TiO_2_ NPs were added, the rates of production of superoxide were the same within uncertainty. The highest O_2_^•−^ production was still observed when 20 μg/mL TiO_2_ NPs were incorporated in the system whatever the sequence of addition of NPs.

## Discussion and Conclusion

Oxide nanoparticles are widely used and their toxicity levels seem to be quite different albeit always related to induction of oxidative stress [27]. Some work has been done on the toxicity of ZnO NPs. A ROS formation enhancement was observed in ZnO-treated liver cells [22,52,53] and on macrophages from wt mice, whereas this formation was impaired in the treated macrophages from the p47^phox-/-^ animals. To our knowledge, this is the only work involving NADPH oxidase. [22]. The use of TiO_2_ NPs has become widespread including in situations where they can be absorbed by living bodies. The photocatalytic activity of TiO_2_ is well known [54], however UVA and visible light do not penetrate inside the body. Thus there is no light exposure and no activation of TiO_2_ NPs by photo-catalysis.

The toxic effects of TiO_2_ NPs seem to be mainly due to indirect production of ROS and therefore to induction of oxidative stress. One of the first studies about interaction between NPs and neutrophils was done in 1988; Hedenborg demonstrated that TiO_2_ induced the production of ROS by human neutrophils [55]. It has also been shown that TiO_2_ NPs can induce oxidative damage to human bronchial epithelial cells in the absence of photoactivation [54,56]. They are known to enhance superoxide production in osteoblasts [16]. TiO_2_ NPs were shown to interact with proteins and enzymes in hepatic tissues, interfering with antioxidant defense mechanisms and leading to generation of ROS [57]. Since NADPH oxidase is a major actor of oxidative stress by producing superoxide ions, it was evident that investigating the effect of TiO_2_ NPs on this enzyme constitutes a relevant issue.

The aim of this paper was to obtain comprehensive information on the interaction of TiO_2_ NPs with the NADPH oxidase. To facilitate such studies, we used a model system that allows performing deeper studies. The different tests performed either with the trimera or with the three separated subunits showed similar results.

It is known that the cytosolic proteins must undergo conformational changes to lead to active enzyme. TiO_2_ NPs have no significant effect on the secondary structure, as shown by the CD spectra (Fig 3). However the fluorescence of the Tryptophan residues is affected by the presence of NPs. Both results are similar to those obtained with fibrinogen [58]. The quenching of fluorescence of the endogenous tryptophans of the trimera indicates that the NPs are probably close to one or several Trp residues and implies that a complex may be formed between the NPs and the cytosolic protein. *In vivo*, it was shown that proteins adsorb on TiO_2_ NPs. In some cases, these NPs induced conformational changes in proteins and affected their functions [12,59,60].

The CD spectra of the trimera in the presence of TiO_2_ NPs and in the presence of AA (Fig 1 and S1 Table [61]) do not exhibit much difference. The Nps have no significant effect on the secondary structure of the trimera as suggested by the CD spectra. Yet, we do not observe any ROS production from NADPH complex in the absence of AA suggesting that TiO_2_ NPs are unable by themselves to activate the enzyme. TEM images showed that membrane fractions are close to TiO_2_ NPs and suggest also an interaction between TiO_2_ NPs and the membranous proteins. Obviously this interaction with the MF does not replace that of the trimera and of AA for activation, and does not prevent AA from having access to the MF, however it might be responsible for the hyperactivation.

Surprisingly, the presence of NPs increases the rate of superoxide anion production (up to 140% of its value without NPs), this effect being dependent on the NPs concentration. They do not interact at a specific step of activation, indicating that their targets are indifferently the membrane fraction as well as the cytosolic proteins and that they can work on the system even when the entire complex is assembled and active. Since the presence of TiO_2_ NPs modifies the AA-dependent activation profile of the enzyme shown in Fig 7, we can postulate that more efficient structure of the NADPH oxidase complex is attained in the presence of NPs. We can exclude a consequence of AA availability due to NPs since the higher NADPH oxidase activity is observed at lower concentration of AA in the presence of NPs than in their absence. This phenomenon cannot be attributed only to an interaction with the sole membrane fraction since the sequence of addition of the NP has no effect on it. An effect on the cytosolic fractions is also likely. Taken together, these facts indicate that the secondary structure of the cytosolic proteins may be conserved and at the same time modifications must have happened to lead to hyperactivation.

Our TEM images showed that NPs remain in the aggregation state even when they are in contact with the proteins. It was demonstrated that particles bigger than 100 nm, can enter phagocytes [62]. Additionally, it was reported that NPs enhance the ability of human neutrophils to exert phagocytosis by a Syk-dependent mechanism [26]. Thus, the TiO_2_ NPs we used can enter cells by phagocytosis and may lead consequently to activation of NADPH oxidase. TiO_2_ NPs aggregates are known to interact with neutrophils. Recent work by SEM [19] showed increased stiffness of the membrane and cell morphology alteration. Our present results indicate that this stiffness would not impede the NADPH oxidase functioning. In conclusion, NADPH oxidase hyper-activation and the subsequent increase in ROS production in the presence of TiO_2_ NPs could be one of the pathways involved in ROS generation by TiO2 NPs, thus participate to their toxicity, which is strongly related to oxidative stress development.

## Supporting Information

S1 Fig Variation of the fluorescence intensity as a function of TiO2 concentration. 340 nm (blue), 440 nm (red). The mixture contained 5 μg/ml (60 nM) trimera and TiO_2_ concentrations of 0, 10, 40, 80 and 100 μg/ml in a final volume of 3 mL of buffer (PBS supplemented with 10 mM MgSO_4_,). The emission spectra were measured using an excitation wavelength of 290 nm as described in the Materials and Methods section. Results are representative of at least three independent experiments.
(TIF)

S2 Fig Fluorescence emission spectra of the tryptophan residues-TiO_2_ NPs suspensions. The solution contains 8μM L-tryptophan and TiO_2_ NPs at the concentrations of 0, 10, 20, 40, 60, 80 and 100 μg/mL in a final volume of 3 mL of buffer (PBS supplemented with 10 mM MgSO_4_). The emission spectra were measured using an excitation wavelength of 290 nm as described in the Materials and Methods section.
(TIF)

S1 Table Analysis of the SRCD spectra of the trimera alone or with cis-AA or with TiO2 NPs. For definition of helixes, sheets and turns, see for instance [60].
(DOCX)

## References

[1] Niederberger M, Pinna N (2009) Metal oxide nanoparticles in organic solvents: synthesis, formation, assembly and application: Springer.

[2] Wiesenthal A, Hunter L, Wang S, Wickliffe J, Wilkerson M (2011) Nanoparticles: small and mighty. Int J Dermatol 50: 247–254. 10.1111/j.1365-4632.2010.04815.x 21342155

[3] Papakostas D, Rancan F, Sterry W, Blume-Peytavi U, Vogt A (2011) Nanoparticles in dermatology. Arch Dermatol Res 303: 533–550. 10.1007/s00403-011-1163-7 21837474

[4] Yuan Y, Ding J, Xu J, Deng J, Guo J (2010) TiO2 nanoparticles co-doped with silver and nitrogen for antibacterial application. J Nanosci Nanotechnol 10: 4868–4874. 21125821 10.1166/jnn.2010.2225

[5] Lucky SS, Muhammad Idris N, Li Z, Huang K, Soo KC, et al (2015) Titania coated upconversion nanoparticles for near-infrared light triggered photodynamic therapy. ACS Nano 9: 191–205. 10.1021/nn503450t 25564723

[6] Zeisser‐Labouèbe M, Vargas A, Delie F (2007) Nanoparticles for photodynamic therapy of cancer. Nanotechnologies for the Life Sciences.

[7] Lagopati N, Kitsiou P, Kontos A, Venieratos P, Kotsopoulou E, et al (2010) Photo-induced treatment of breast epithelial cancer cells using nanostructured titanium dioxide solution. Journal of Photochemistry and Photobiology A: Chemistry 214: 215–223.

[8] Dransfield G (2000) Inorganic sunscreens. Radiation protection dosimetry 91: 271–273.

[9] Thomas T, Thomas K, Sadrieh N, Savage N, Adair P, et al (2006) Research strategies for safety evaluation of nanomaterials, part VII: evaluating consumer exposure to nanoscale materials. Toxicol Sci 91: 14–19. 16476686 10.1093/toxsci/kfj129

[10] Bermudez E, Mangum JB, Wong BA, Asgharian B, Hext PM, et al (2004) Pulmonary responses of mice, rats, and hamsters to subchronic inhalation of ultrafine titanium dioxide particles. Toxicol Sci 77: 347–357. 14600271 10.1093/toxsci/kfh019

[11] Wang J, Zhou G, Chen C, Yu H, Wang T, et al (2007) Acute toxicity and biodistribution of different sized titanium dioxide particles in mice after oral administration. Toxicol Lett 168: 176–185. 17197136 10.1016/j.toxlet.2006.12.001

[12] Gheshlaghi ZN, Riazi GH, Ahmadian S, Ghafari M, Mahinpour R (2008) Toxicity and interaction of titanium dioxide nanoparticles with microtubule protein. Acta Biochim Biophys Sin (Shanghai) 40: 777–782. 18776989

[13] Barnard AS (2010) One-to-one comparison of sunscreen efficacy, aesthetics and potential nanotoxicity. Nat Nanotechnol 5: 271–274. 10.1038/nnano.2010.25 20208548

[14] Buzea C, Pacheco II, Robbie K (2007) Nanomaterials and nanoparticles: sources and toxicity. Biointerphases 2: MR17–MR71. 20419892 10.1116/1.2815690

[15] Manke A, Wang L, Rojanasakul Y (2013) Mechanisms of Nanoparticle-Induced Oxidative Stress and Toxicity. BioMed Research International 2013: 15. 10.1155/2013/942916 PMC3762079 24027766

[16] Niska K, Pyszka K, Tukaj C, Wozniak M, Radomski MW, et al (2015) Titanium dioxide nanoparticles enhance production of superoxide anion and alter the antioxidant system in human osteoblast cells. Int J Nanomedicine 10: 1095–1107. 10.2147/IJN.S73557 25709434 PMC4327568

[17] Skocaj M, Filipic M, Petkovic J, Novak S (2011) Titanium dioxide in our everyday life; is it safe? Radiol Oncol 45: 227–247. 10.2478/v10019-011-0037-0 22933961 PMC3423755

[18] Shi H, Magaye R, Castranova V, Zhao J (2013) Titanium dioxide nanoparticles: a review of current toxicological data. Part Fibre Toxicol 10: 15 10.1186/1743-8977-10-15 23587290 PMC3637140

[19] da Rosa EL (2013) Kinetic effects of TiO2 fine particles and nanoparticles aggregates on the nanomechanical properties of human neutrophils assessed by force spectroscopy. BMC Biophys 6: 11 10.1186/2046-1682-6-11 23957965 PMC3766645

[20] Huang CC, Aronstam RS, Chen DR, Huang YW (2010) Oxidative stress, calcium homeostasis, and altered gene expression in human lung epithelial cells exposed to ZnO nanoparticles. Toxicol In Vitro 24: 45–55. 10.1016/j.tiv.2009.09.007 19755143

[21] Knaapen AM, Borm PJ, Albrecht C, Schins RP (2004) Inhaled particles and lung cancer. Part A: Mechanisms. Int J Cancer 109: 799–809. 15027112 10.1002/ijc.11708

[22] Wilhelmi V, Fischer U, Weighardt H, Schulze-Osthoff K, Nickel C, et al (2013) Zinc oxide nanoparticles induce necrosis and apoptosis in macrophages in a p47phox- and Nrf2-independent manner. PLoS One 8: e65704 10.1371/journal.pone.0065704 23755271 PMC3670863

[23] Goncalves DM, de Liz R, Girard D (2011) Activation of neutrophils by nanoparticles. ScientificWorldJournal 11: 1877–1885. 10.1100/2011/768350 22125444 PMC3217611

[24] Goncalves DM, Chiasson S, Girard D (2010) Activation of human neutrophils by titanium dioxide (TiO2) nanoparticles. Toxicol In Vitro 24: 1002–1008. 10.1016/j.tiv.2009.12.007 20005940

[25] Jovanovic B, Anastasova L., Rowe E. W., Zhang Y., Clapp A. R. & Palic D. (2011) Effects of nanosized titanium dioxide on innate immune system of fathead minnow (Pimephales promelas Rafinesque, 1820). Ecotoxicology and environmental safety 74: 675–683 10.1016/j.ecoenv.2010.10.017 21035856

[26] Babin K, Goncalves DM, Girard D (2015) Nanoparticles enhance the ability of human neutrophils to exert phagocytosis by a Syk-dependent mechanism. Biochim Biophys Acta 1850: 2276–2282. 10.1016/j.bbagen.2015.08.006 26277637

[27] Xia T, Kovochich M, Brant J, Hotze M, Sempf J, et al (2006) Comparison of the abilities of ambient and manufactured nanoparticles to induce cellular toxicity according to an oxidative stress paradigm. Nano Lett 6: 1794–1807. 16895376 10.1021/nl061025k

[28] Nauseef WM (2007) How human neutrophils kill and degrade microbes: an integrated view. Immunological Reviews 219: 88–102. 17850484 10.1111/j.1600-065X.2007.00550.x

[29] Segal AW (2005) How neutrophils kill microbes. Annu Rev Immunol 23: 197–223. 15771570 10.1146/annurev.immunol.23.021704.115653 PMC2092448

[30] Babior BM (1984) Oxidants from phagocytes: agents of defense and destruction. Blood 64: 959–966. 6386073

[31] Sumimoto H (2008) Structure, regulation and evolution of Nox-family NADPH oxidases that produce reactive oxygen species. FEBS Journal 275: 3249–3277. 10.1111/j.1742-4658.2008.06488.x 18513324

[32] Valencia A, Kochevar IE (2007) Nox1-based NADPH oxidase is the major source of UVA-induced reactive oxygen species in human keratinocytes. Journal of Investigative Dermatology 128: 214–222. 17611574 10.1038/sj.jid.5700960

[33] El-Benna J, Dang PM, Gougerot-Pocidalo MA, Marie JC, Braut-Boucher F (2009) p47phox, the phagocyte NADPH oxidase/NOX2 organizer: structure, phosphorylation and implication in diseases. Exp Mol Med 41: 217–225. 10.3858/emm.2009.41.4.058 19372727 PMC2679237

[34] Sheppard FR, Kelher MR, Moore EE, McLaughlin NJ, Banerjee A, et al (2005) Structural organization of the neutrophil NADPH oxidase: phosphorylation and translocation during priming and activation. J Leukoc Biol 78: 1025–1042. 16204621 10.1189/jlb.0804442

[35] Bedard K, Krause KH (2007) The NOX family of ROS-generating NADPH oxidases: physiology and pathophysiology. Physiol Rev 87: 245–313. 17237347 10.1152/physrev.00044.2005

[36] Babior BM (2004) NADPH oxidase. Current Opinion in Immunology 16: 42–47. 14734109 10.1016/j.coi.2003.12.001

[37] Nauseef W (2004) Assembly of the phagocyte NADPH oxidase. Histochemistry and Cell Biology 122: 277–291. 15293055 10.1007/s00418-004-0679-8

[38] Raad H, Paclet MH, Boussetta T, Kroviarski Y, Morel F, et al (2009) Regulation of the phagocyte NADPH oxidase activity: phosphorylation of gp91phox/NOX2 by protein kinase C enhances its diaphorase activity and binding to Rac2, p67phox, and p47phox. FASEB J 23: 1011–1022. 10.1096/fj.08-114553 19028840 PMC2660639

[39] Baciou L, Erard M, Dagher MC, Bizouarn T (2009) The cytosolic subunit p67phox of the NADPH-oxidase complex does not bind NADPH. FEBS Lett 583: 3225–3229. 10.1016/j.febslet.2009.09.011 19751728

[40] Ostuni MA, Gelinotte M, Bizouarn T, Baciou L, Houee-Levin C (2010) Targeting NADPH-oxidase by reactive oxygen species reveals an initial sensitive step in the assembly process. Free Radic Biol Med 49: 900–907. 10.1016/j.freeradbiomed.2010.06.021 20600833

[41] Karimi G, Houee Levin C, Dagher MC, Baciou L, Bizouarn T (2014) Assembly of phagocyte NADPH oxidase: A concerted binding process? Biochim Biophys Acta 1840: 3277–3283. 10.1016/j.bbagen.2014.07.022 25108064

[42] Souabni H, Machillot P, Baciou L Contribution of lipid environment to NADPH oxidase activity: Influence of sterol. Biochimie. 10.1016/j.biochi.2014.10.006 25448770

[43] Souabni H, Thoma V, Bizouarn T, Chatgilialoglu C, Siafaka-Kapadai A, et al (2012) trans Arachidonic acid isomers inhibit NADPH-oxidase activity by direct interaction with enzyme components. Biochim Biophys Acta 1818: 2314–2324. 10.1016/j.bbamem.2012.04.018 22580228

[44] Berdichevsky Y, Mizrahi A, Ugolev Y, Molshanski-Mor S, Pick E (2007) Tripartite chimeras comprising functional domains derived from the cytosolic NADPH oxidase components p47phox, p67phox, and Rac1 elicit activator-independent superoxide production by phagocyte membranes: an essential role for anionic membrane phospholipids. J Biol Chem 282: 22122–22139. 17548354 10.1074/jbc.M701497200

[45] Masoud R, Bizouarn T., Houée-Levin C. (2014) Cholesterol: A modulator of the phagocyte NADPH oxidase activity - A cell-free study. Redox Biology 3: 16–24. 10.1016/j.redox.2014.10.001 25462061 PMC4221629

[46] Marucco A, Catalano F, Fenoglio I, Turci F, Martra G, et al (2015) Possible Chemical Source of Discrepancy between in Vitro and in Vivo Tests in Nanotoxicology Caused by Strong Adsorption of Buffer Components. Chem Res Toxicol. 10.1021/tx500366a 25564874

[47] Akasaki T, Koga H, Sumimoto H (1999) Phosphoinositide 3-kinase-dependent and -independent activation of the small GTPase Rac2 in human neutrophils. J Biol Chem 274: 18055–18059. 10364257 10.1074/jbc.274.25.18055

[48] Swain SD, Helgerson SL, Davis AR, Nelson LK, Quinn MT (1997) Analysis of activation-induced conformational changes in p47phox using tryptophan fluorescence spectroscopy. J Biol Chem 272: 29502–29510. 9368011 10.1074/jbc.272.47.29502

[49] Micsonai A, Wien F, Kernya L, Lee YH, Goto Y, et al (2015) Accurate secondary structure prediction and fold recognition for circular dichroism spectroscopy. Proc Natl Acad Sci U S A 112: E3095–3103. 10.1073/pnas.1500851112 26038575 PMC4475991

[50] Pick E, Bromberg Y, Shpungin S, Gadba R (1987) Activation of the superoxide forming NADPH oxidase in a cell-free system by sodium dodecyl sulfate. Characterization of the membrane-associated component. J Biol Chem 262: 16476–16483. 2824496

[51] Shiose A, Sumimoto H (2000) Arachidonic acid and phosphorylation synergistically induce a conformational change of p47phox to activate the phagocyte NADPH oxidase. J Biol Chem 275: 13793–13801. 10788501 10.1074/jbc.275.18.13793

[52] Sharma V, Singh P, Pandey AK, Dhawan A (2012) Induction of oxidative stress, DNA damage and apoptosis in mouse liver after sub-acute oral exposure to zinc oxide nanoparticles. Mutat Res 745: 84–91. 10.1016/j.mrgentox.2011.12.009 22198329

[53] Sharma V, Anderson D, Dhawan A (2012) Zinc oxide nanoparticles induce oxidative DNA damage and ROS-triggered mitochondria mediated apoptosis in human liver cells (HepG2). Apoptosis 17: 852–870. 10.1007/s10495-012-0705-6 22395444

[54] Jovanovic B (2015) Review of titanium dioxide nanoparticle phototoxicity: Developing a phototoxicity ratio to correct the endpoint values of toxicity tests. Environ Toxicol Chem 34: 1070–1077. 10.1002/etc.2891 25640001 PMC5008198

[55] Hedenborg M (1988) Titanium dioxide induced chemiluminescence of human polymorphonuclear leukocytes. Int Arch Occup Environ Health 61: 1–6. 3198275 10.1007/BF00381600

[56] Gurr JR, Wang AS, Chen CH, Jan KY (2005) Ultrafine titanium dioxide particles in the absence of photoactivation can induce oxidative damage to human bronchial epithelial cells. Toxicology 213: 66–73. 15970370 10.1016/j.tox.2005.05.007

[57] Alarifi S, Ali D, Al-Doaiss AA, Ali BA, Ahmed M, et al (2013) Histologic and apoptotic changes induced by titanium dioxide nanoparticles in the livers of rats. Int J Nanomedicine 8: 3937–3943. 10.2147/IJN.S47174 24143098 PMC3798149

[58] Wang C, Li Y (2012) Interaction and nanotoxic effect of TiO2 nanoparticle on fibrinogen by multi-spectroscopic method. Science of The Total Environment 429: 156–160. 10.1016/j.scitotenv.2012.03.048 22607744

[59] Allouni ZE, Gjerdet NR, Cimpan MR, Hol PJ (2015) The effect of blood protein adsorption on cellular uptake of anatase TiO2 nanoparticles. Int J Nanomedicine 10: 687–695. 10.2147/IJN.S72726 25632230 PMC4304597

[60] Simon-Vazquez R, Lozano-Fernandez T, Peleteiro-Olmedo M, Gonzalez-Fernandez A (2014) Conformational changes in human plasma proteins induced by metal oxide nanoparticles. Colloids Surf B Biointerfaces 113: 198–206. 10.1016/j.colsurfb.2013.08.047 24095988

[61] Voet D, Voet J. (2011) Biochemistry 4th edition New York: Wiley.

[62] Aderem A, Underhill DM (1999) Mechanisms of phagocytosis in macrophages. Annu Rev Immunol 17: 593–623. 10358769 10.1146/annurev.immunol.17.1.593

