# Supplementary figures and images for: Titanium Dioxide Nanoparticles Increase Superoxide Anion Production by Acting on NADPH Oxidase
Source: PLoS One. 2015 Dec 29;10(12):e0144829. doi: 10.1371/journal.pone.0144829 (PMC4699827; doi:10.1371/journal.pone.0144829)

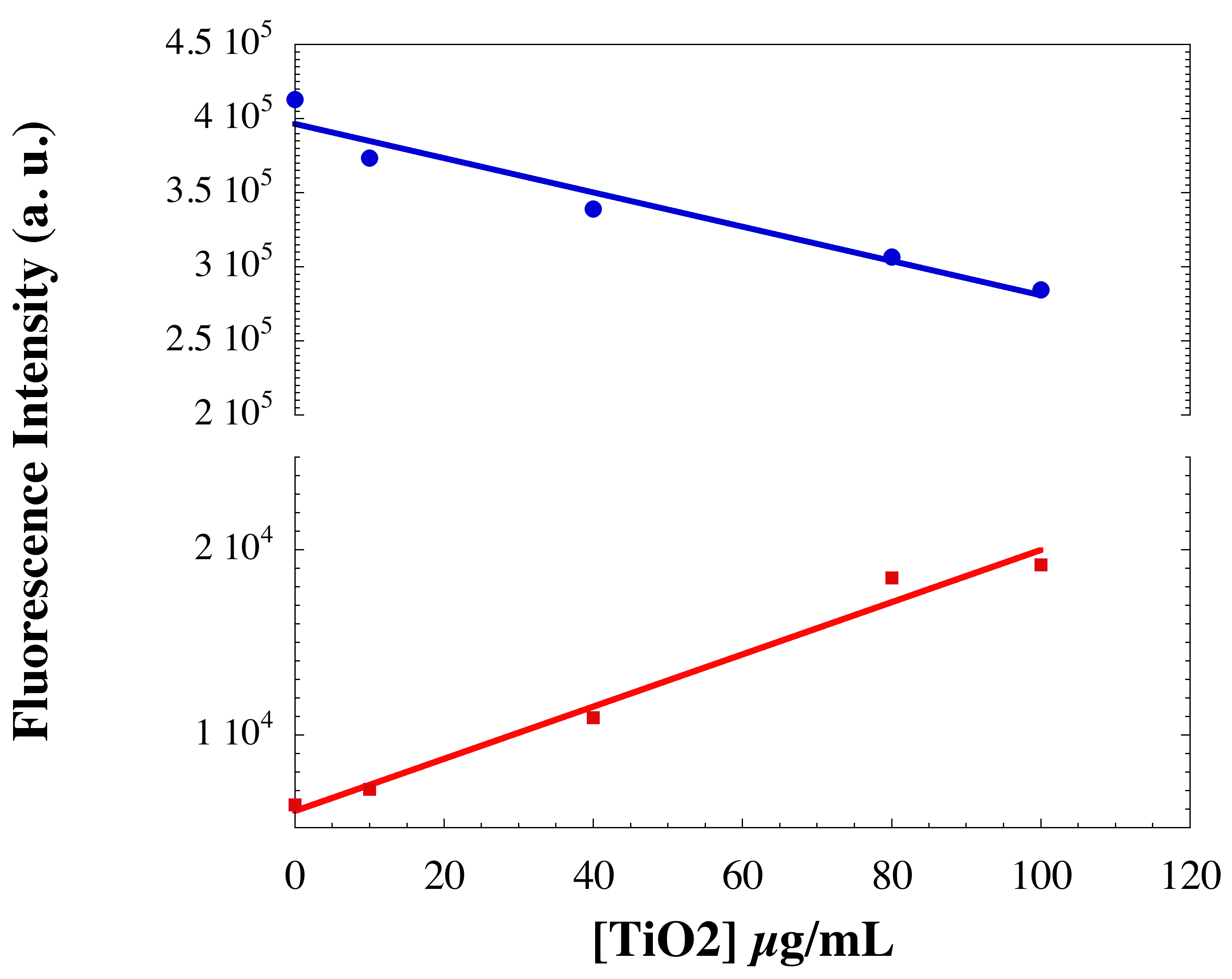

Supplement: S1 Fig — 340 nm (blue), 440 nm (red). The mixture contained 5 μg/ml (60 nM) trimera and TiO2 concentrations of 0, 10, 40, 80 and 100 μg/ml in a final volume of 3 mL of buffer (PBS supplemented with 10 mM MgSO4,). The emission spectra were measured using an excitation wavelength of 290 nm as described in the Materials and Methods section. Results are representative of at least three independent experiments. (TIF) [file pone.0144829.s001.tif]

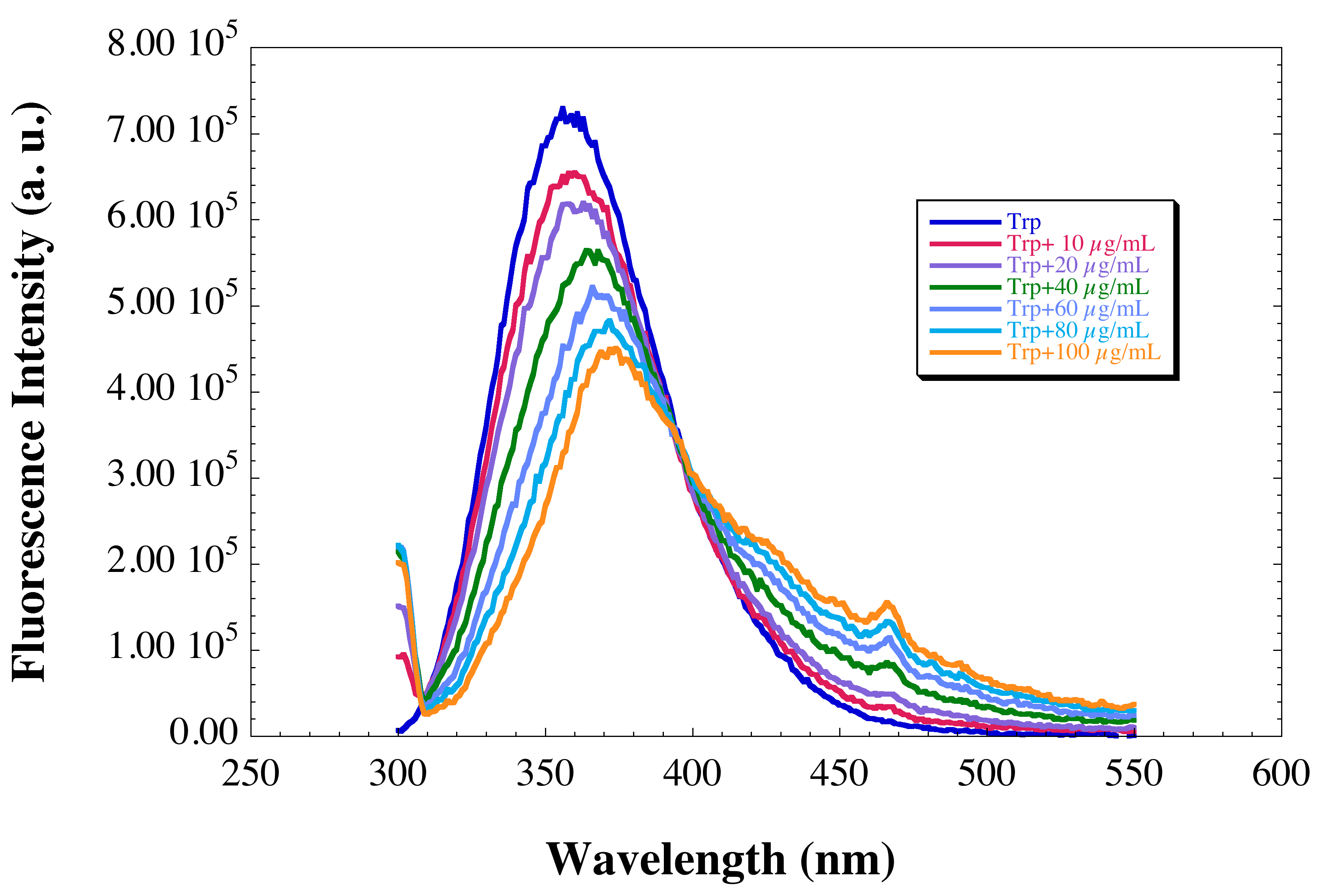

Supplement: S2 Fig — The solution contains 8μM L-tryptophan and TiO2 NPs at the concentrations of 0, 10, 20, 40, 60, 80 and 100 μg/mL in a final volume of 3 mL of buffer (PBS supplemented with 10 mM MgSO4). The emission spectra were measured using an excitation wavelength of 290 nm as described in the Materials and Methods section. (TIF) [file pone.0144829.s002.tif]
