# Supplementary material for: Titanium Dioxide Nanoparticles Increase Superoxide Anion Production by Acting on NADPH Oxidase
Source: PLoS One. 2015 Dec 29;10(12):e0144829. doi: 10.1371/journal.pone.0144829 (PMC4699827; doi:10.1371/journal.pone.0144829)
Supplement: S1 Table — For definition of helixes, sheets and turns, see for instance [60]. (DOCX) [file pone.0144829.s003.docx]

Table S1: analysis of the SRCD spectra of the trimera in solution or with cis-AA or with TiO2 NPs.

|  | α-helix | β-sheets | Turns | Other |
| --- | --- | --- | --- | --- |
| Trimera | 4.1 | 40.5 | 13.8 | 41.7 |
| Trimera + cis-AA 300µM | 2.5 | 42.0 | 13.7 | 41.8 |
| Trimera + TiO2 NPs PC500 | 3.1 | 40.1 | 13.9 | 42.9 |
